# Supplementary material for: Acetyl-CoA Carboxylase (ACC) Inhibitor, CP640186, Effectively Inhibited Dengue Virus (DENV) Infection via Regulating ACC Phosphorylation
Source: Molecules. 2022 Dec 5;27(23):8583. doi: 10.3390/molecules27238583 (PMC9739643; doi:10.3390/molecules27238583)
Supplement: Supplementary file 1 [file molecules-27-08583-s001.zip › molecules-2020545-supplementary.pdf]

# Supplymental Figures

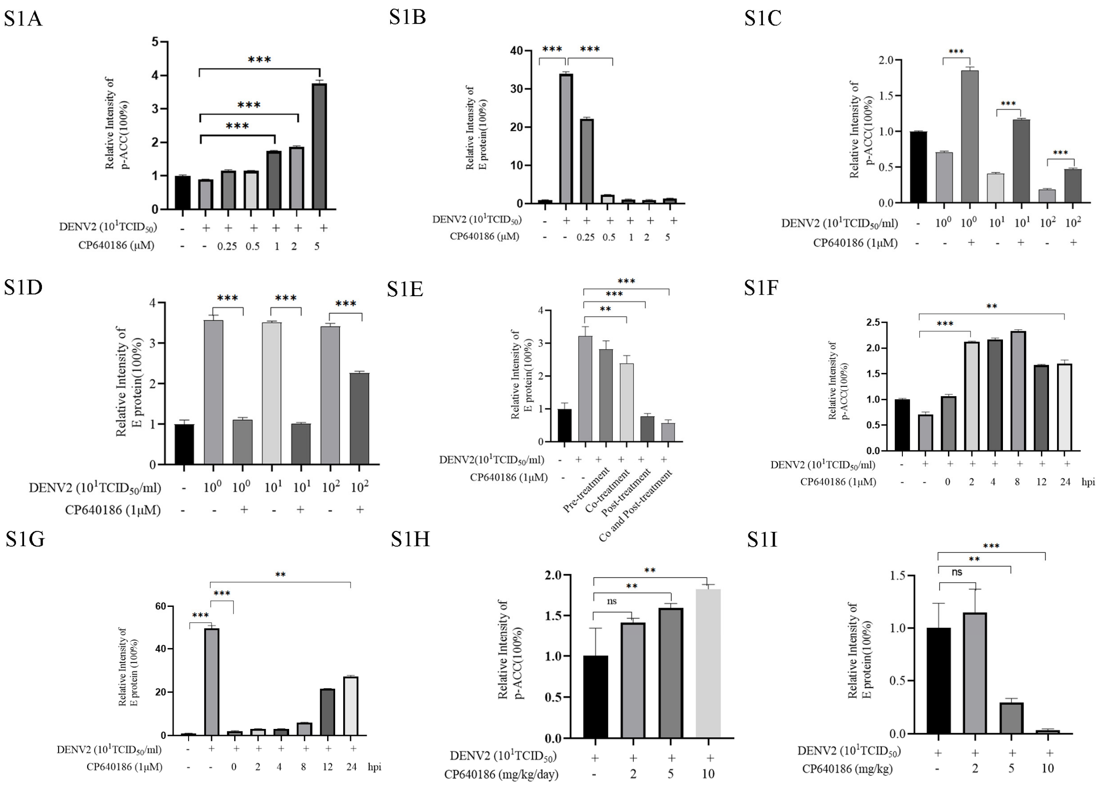


**Figure S1.** The quantification of western bloting results. (A,B) The quantification of the P-ACC and E protein level in Figure 2A. (C,D) The quantification of the P-ACC and E protein level in Figure 3A. (E) The quantification of E protein level in Figure 4B. (F,G) The quantification of the P-ACC and E protein level in Figure 4F. (H,I) The quantification of the P-ACC and E protein level in Figure 5E. ** indicates $p < 0.01$. *** indicates $p < 0.001$.

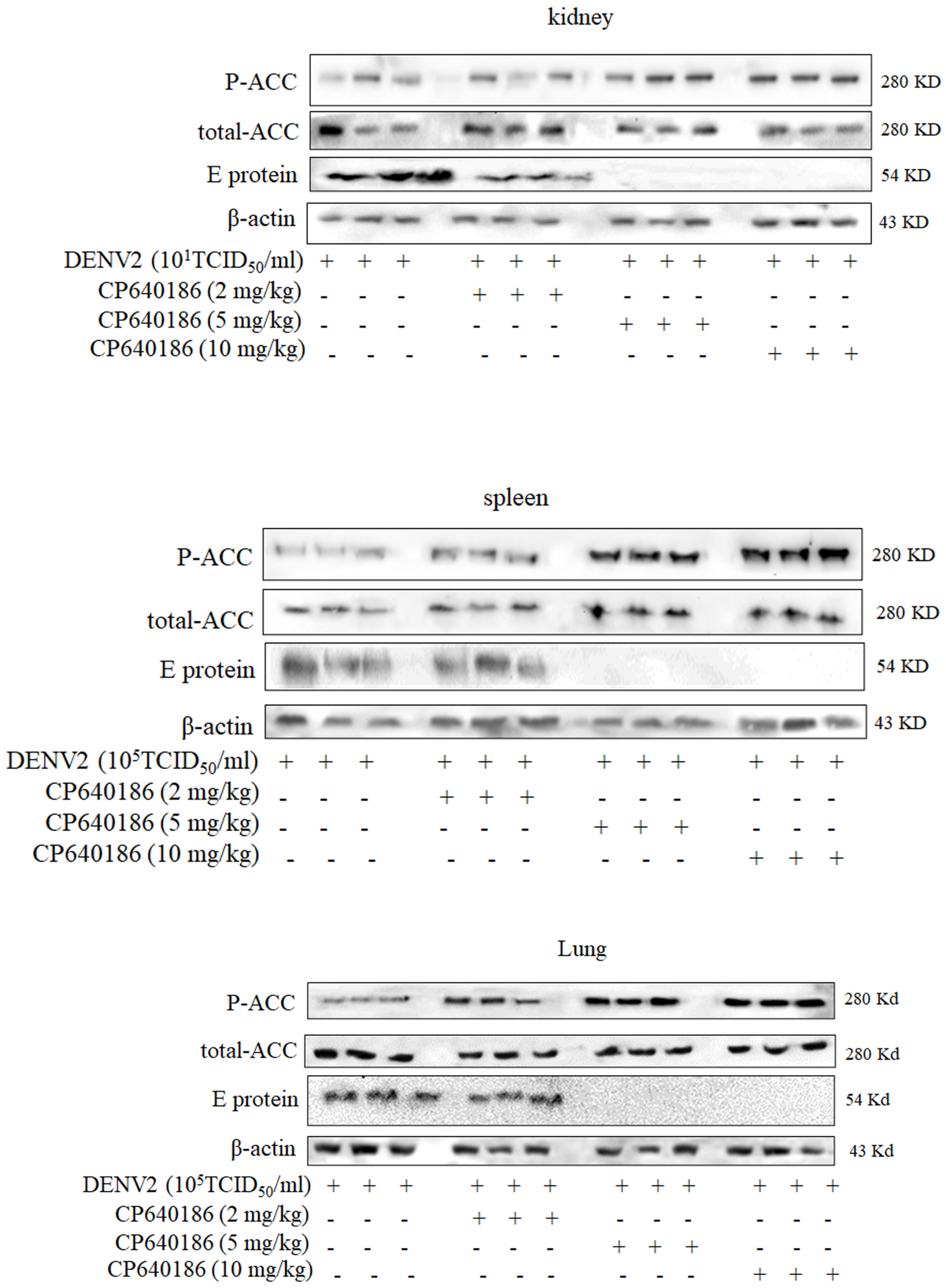


**Figure S2.** The western bloting resuts in mice tissues.
